# Practical Department

**Published:** 1902-07-26

**Authors:** 


### PRACTICAL DEPARTMENT.

### HOSPITAL FLOORS.

A correspondent writes: "Will you kindly tell me the best way to polish a ward floor. It is pitch-pine wood, and has had a coating of linseed oil, and is being rubbed with beeswax and turpentine, but the polish is not satisfactory?"

[Pitch pine is not hard enough for hospital floors. There would appear to be no remedy but new floors. Try scrubbing in lieu of polishing; then cover with linoleum if the surface of floors warrants it—Ed. The Hospital.]

